# Secular Trends in Habitual Physical Activities of Mozambican Children and Adolescents from Maputo City

**DOI:** 10.3390/ijerph111010940

**Published:** 2014-10-21

**Authors:** Fernanda Karina dos Santos, José A. R. Maia, Thayse Natacha Q. F. Gomes, Timóteo Daca, Aspacia Madeira, Albertino Damasceno, Peter T. Katzmarzyk, António Prista

**Affiliations:** 1 CIFI^2^D, Kinanthropometry Lab, Faculty of Sport, University of Porto, Rua Dr. Plácido Costa 91, Porto 4200-450, Portugal; E-Mails: fernandak.santos@hotmail.com (F.K.S.); jmaia@fade.up.pt (J.A.R.M.); thayse_natacha@hotmail.com (T.N.Q.F.G.); 2 CAPES Foundation, Ministry of Education of Brazil, Brasília, DF 70040-020, Brazil; 3 Faculty of Physical Education and Sports, Pedagogical University of Maputo, Avenida Eduardo Mondlane 955, C.P. 2017, Maputo, Mozambique; E-Mails: dacajunior@gmail.com (T.D.); aspacia.madeira@gmail.com (A.M.); 4 Faculty of Medicine, University Eduardo Mondlane, Maputo, Avenida Salvador Allende 702, C.P. 257 Maputo, Mozambique; E-Mail: tino_7117@hotmail.com; 5 Pennington Biomedical Research Center, Louisiana State University, 6400 Perkins Rd., Baton Rouge, LA 70808, USA; E-Mail: Peter.Katzmarzyk@pbrc.edu

**Keywords:** secular trends, physical activity, youth, Mozambique

## Abstract

Social and economic changes occurring in the last two decades in Mozambique may have induced lifestyle changes among youth. This study aimed to document secular changes in habitual physical activities of Mozambican youth between 1992, 1999 and 2012. A total of 3393 youth (eight–15 years), were measured in three different time periods (1992, 1999, 2012). Habitual physical activity (PA) was estimated with a questionnaire, including items related to household chores, sport participation, traditional games and walking activities. Biological maturation was assessed. Analysis of Covariance (ANCOVA) was used to compare mean differences in PA across the years. Significant decreases between 1992–1999 and 1992–2012 were observed for boys in household chores, games and walking, and a significant decline between 1999 and 2012 was found in sport participation.Among girls, a significant and consistent decline (1992 > 1999 > 2012) was observed for household chores, a decline between 1992–1999 and 1992–2012 for games and walking, and a significant increase between 1992 and 1999 in sport participation. In general, a negative secular trend was found in habitual PA among Mozambican youth. Interventions aimed at increasing PA represent important educational and public health opportunities.

## 1. Introduction

It has been suggested that a decline in physical activity (PA) levels has occurred in recent decades among the pediatric population [1]. However, Ekelund *et al*. [2] pointed out in a recent review that in light of the well-known limitations of self-reported PA, and variability in age, sample size, and characteristics of the available samples, that such claims regarding this decline may not be sufficiently supported. Further, they reported that about 30%–40% of youth are sufficiently active based on current PA guidelines. However, there is consistent evidence that the urbanization process is associated with greater mechanization and socioe-conomic and demographic changes which are associated with the adoption of a lifestyle characterized by lower energy requirements at work, less dependence on walking/commuting to school or work, and the widespread adoption of sedentary activities [3].

In developing countries and subsistence societies, household chores, active transportation and labor activities are important contributors to total daily energy expenditure, more so than leisure activities [4]. In addition, children are usually engaged in strenuous and skilled household chores such as pounding grain, carrying water, cutting wood and agriculture [4]. Since within these transitional societies the consequences of growing urbanization is a continuous process, with changes in urban environments, technological advancements, industrialization, and material prosperity, in all likelihood these transformations lead to changes in youth lifestyles, with consequences for their daily habitual PA [3].

The investigation of secular trends in PA among youth has occurred mainly in developed countries [5]. In developing countries such information is scarce, particularly in Africa [6]. As several African countries are experiencing economic and demographic transitions linked with uninterrupted changes in urbanization, it is important to study secular trends in PA in these societies to better understand the changes in relation to social, economic and cultural changes [3,6].

In Mozambique, social and economic changes occurring in the last two decades, since the end of the War (1992), provided enhancements in the quality of life and well-being of the population [7]. As a consequence of this transition, changes in lifestyle, including the increased use of personal computers and videogames, as well as the rise in overweight and obesity prevalence were observed [8,9]. This study aimed to document the secular changes in habitual PA among Mozambican boys and girls between 1992, 1999 and 2012, comprising a period from the end of the War until a period of peace and economic growth.

## 2. Methods

### 2.1. Mozambique

Mozambique is a country located in Southeast Africa, bordered by the Indian Ocean, Tanzania, Malawi, Zambia, Zimbabwe, Swaziland, and South Africa. With an estimated total population (in 2012) of approximately 25 million [10], and a population growth rate of 2.4% during the last two decades, Mozambique has experienced significant economic growth as a consequence of changes occurring since the end of the war. Notwithstanding this rapid growth, Mozambique is still an underdeveloped country, with significant social disparities within its population.

The city of Maputo, the capital and the largest city of Mozambique, is located in the south of the country, on the west bank of the Maputo Bay and since 1980 has the status of province [11]. The city has about 1,194,121 inhabitants, with a life expectancy of approximately 57.5 years; the population density is 3443.6 inhabitants/km^2^ and the population growth rate is 1.37% [12,13].

This study is part of the “Human Biological Variability-Implications for Physical Education, Sports, Preventive Medicine and Public Health” research project [14] that aims to describe the patterns of human variability in growth, biological maturation and development of Mozambican youth, and to understand the role of genetic and environmental factors in the variability of these indicators in this population. This project started in 1992, and has had three periods of data collection to date in Maputo: 1992, 1999 and 2012.

### 2.2. Sample

The sample for the present study comprises children and adolescents aged eight–15 years, assessed in each time period, following a three-stage cluster sampling design (areas, schools and students), with youth been enlisted from the same schools, from urban or suburban areas of Maputo [15]. All children and adolescents involved in the project had a written consent signed by parents or legal guardians, and those without a signed consent form, with chronic diseases, physical handicaps or psychological disorders were excluded during sample selection and/or data screening. Moreover, for the present study, those children and adolescents younger than eight years or older than 15 years, and those with missing information (458 subjects) were excluded from analysis. Data were missing at random, and no statistically significant differences were identified between those with missing data and those with complete data (analysis not shown). The final sample was distributed as follows: 498 subjects in 1992 (232 boys, 266 girls); 1347 subjects in 1999 (620 boys, 727 girls), and 1187 subjects in 2012 (569 boys, 618 girls), comprising 3393 subjects (1568 boys, 1825 girls). The study protocol was approved by the Mozambican National Bioethics Committee.

### 2.3. Physical Activity

Habitual PA was estimated with a questionnaire which was developed and validated for Mozambican youth [16]. It includes questions related to four domains: household chores, sport participation, games and walking activities. Subjects were asked how many times they performed all the activities included in the questionnaire in a typical week. An activity score for each domain was computed based on the sum of the products of the estimated energy cost of each activity (expressed in METs) multiplied by the number of times per week the activity was performed; further, a final total activity coefficient (AC) was calculated as the sum of the activity score derived from each domain. The questionnaire was administered in an individually-based interview by trained technicians.

### 2.4. Biological Maturation

Biological maturity was assessed using procedures described by Tanner and Whitehouse [17], and children were classified according to their secondary sexual characteristics (pubic hair stages). Trained observers from the same gender rated all children/adolescents.

### 2.5. Statistical Analysis

Exploratory analyses and data quality checks were systematically performed in Epi Info software, version 3.5.3 prior to all subsequent data analysis. Basic descriptive statistics were computed, and analysis of covariance (ANCOVA) adjusted for age, biological maturation and BMI was used to compare differences in PA values (for each domain and for AC) across the survey years. Multicollinearity checks were made when including age and sexual maturation as covariates in ANCOVA as suggested by Tabachnick and Fidell [18]. Bonferroni adjustments were applied to all comparisons. Trends across study years were tested using a polynomial contrast. SPSS 20.0 was used for all analyses, and the significance level was set at 5%.

## 3. Results

Table 1 shows descriptive statistics for each variable, by gender and year. Since there was a significant interaction between gender and assessment year for almost all PA domains (results not shown), separate analyses were performed for boys and girls.

**Table 1:** Descriptive statistics by gender and year.

Variables	Boys			Girls		
	1992	1999	2012	1992	1999	2012
	M ± SD	M ± SD	M ± SD	M ± SD	M ± SD	M ± SD
N	232	620	569	266	727	618
Age	11.53 ± 2.14	12.24 ± 2.08	11.35 ± 2.19	11.41 ± 2.26	12.07 ± 2.20	11.26 ± 2.20
Maturity Status *	1.00 (1)	1.00 (2)	1.00 (1)	1.00 (2)	2.00 (2)	2.00 (2)
BMI	16.12 ± 1.94	16.65 ± 2.39	17.46 ± 2.87	17.29 ± 2.74	17.92 ± 3.20	18.55 ± 3.81
Household chores	17.31 ± 12.05	12.72 ± 10.63	12.53 ± 11.54	25.18 ± 14.83	22.21 ± 13.85	15.06 ± 11.73
Games	28.96 ± 16.60	22.23 ± 14.61	25.53 ± 18.19	37.22 ± 20.92	28.85 ± 19.17	28.04 ± 17.86
Sports	21.07 ± 14.16	23.41 ± 12.09	20.59 ± 14.97	11.93 ± 12.64	14.58 ± 12.05	12.03 ± 11.02
Walking	50.01 ± 19.70	41.95 ± 15.37	39.41 ± 15.60	48.19 ± 18.18	39.07 ± 15.45	38.82 ± 16.17
AC (Total)	117.36 ± 36.19	100.32 ± 30.62	98.06 ± 39.31	122.52 ± 38.23	104.71 ± 34.24	93.95 ± 33.64

Note: * Median (interquartile range).

ANCOVA results (means ± standard errors, F and *p*-values) for each activity domain score, as well as AC, for boys and girls (controlling for age, maturity status and BMI), are presented on Table 2. For boys, a decrease from 1992 to 2012, with significant differences between 1992–1999 and 1992–2012, but not between 1999 and 2012, were observed in almost all PA domains - household chores, games, and walking as well as for the AC. However, in the sport participation domain, a significant decline was only observed between 1999 and 2012. Among girls, the results suggest a progressive and consistent decline (1992 > 1999 > 2012) in household chores and AC; however, for the games and walking domains, girls in 1992 had higher scores than those in 1999 and 2012, and a significant decline was found between 1999 and 2012 for the sports domain. It is relevant to highlight that since PA changes with age, and different activities are performed by younger and older children, significant age effects (*p* < 0.05) were found for almost all PA domains in both genders (except for household chores in boys) as well for AC, with values ranging from −1.312 to 3.575. The negative coefficient was observed only in the games domain (for both boys and girls), meaning that with increasing age the MET scores in household chores (for girls), sports, walking and AC also increase.

Table 2 also shows, for boys, significant linear and non-linear trends for household chores (*p* < 0.001 for linear trend; *p* = 0.001 for non-linear trend), games (*p* < 0.05), walking (*p* < 0.001) and AC (*p* < 0.001), and significant non-linear trend for sports (*p* = 0.004). While for girls, it was observed significant linear and non-linear trends for games (*p* < 0.001 for linear trend; *p* = 0.003 for non-linear trend), walking (*p* < 0.001) and AC (*p* < 0.001 for linear trend; *p* = 0.002 for non-linear trend), significant linear trend for household chores (*p* < 0.001) and significant non-linear trend for sports (*p* = 0.003).

**Table 2:** Activity domain scores and total AC (adjusted for age, maturity status and BMI) for boys and girls (eight–15 years), by year of assessment, as well as pairwise comparisons.

Variables	1992	1999	2012	*F*	*p-*value	*Partial Eta Squared*	*p* for Trend	*Pairwise comparisons*
	M ± SE	M ± SE	M ± SE					
Boys	*n* = 232	*n* = 620	*n* = 569					
Household chores	17.36 ± 0.73	12.75 ± 0.60	12.49 ± 0.49	16.962	<0.001	0.023	L < 0.001; NL = 0.001	1992 > 1999; 1992 > 2012
Games	28.45 ± 1.05	23.14 ± 0.65	24.74 ± 0.69	9.284	<0.001	0.013	L = 0.004; NL < 0.001	1992 > 1999; 1992 > 2012
Sports	21.41 ± 0.90	23.26 ± 0.56	20.63 ± 0.59	5.280	0.005	0.007	L = 0.469; NL = 0.004	1999 > 2012
Walking	50.40 ± 1.05	41.22 ± 0.65	40.05 ± 0.69	36.315	<0.001	0.049	L < 0.001; NL < 0.001	1992 > 1999; 1992 > 2012
AC (Total)	117.61 ± 2.32	100.36 ± 1.44	97.90 ± 1.52	26.729	<0.001	0.036	L < 0.001; NL < 0.001	1992 > 1999; 1992 > 2012
**Girls**	***n* = 266**	***n* = 727**	***n* = 618**					
Household chores	24.80 ± 0.84	21.62 ± 0.50	15.92 ± 0.57	41.534	<0.001	0.049	L < 0.001; NL = 0.070	1992 > 1999 > 2012
Games	34.50 ± 1.15	28.95 ± 0.69	29.09 ± 0.78	9.624	<0.001	0.012	L < 0.001; NL = 0.003	1992 > 1999; 1992 > 2012
Sports	12.36 ± 0.72	14.15 ± 0.44	12.35 ± 0.49	4.604	0.010	0.006	L = 0.989; NL = 0.003	1999 > 2012
Walking	48.46 ± 1.00	38.26 ± 0.60	39.65 ± 0.69	40.091	<0.001	0.048	L < 0.001; NL<0.001	1992 > 1999; 1992 > 2012
AC (Total)	120.12 ± 2.19	102.99 ± 1.31	97.00 ± 1.50	36.025	<0.001	0.043	L < 0.001; NL = 0.002	1992 > 1999 > 2012

Notes: L = Linear trend; NL = No linear trend.

## 4. Discussion

The purpose of this study was to identify secular changes in habitual PA of Mozambican boys and girls, embracing a period of twenty years where Mozambique went through economic and social changes as a consequence of the end of the War. This transitional period had consequences in Mozambicans’ lives with improvements in their quality of life as well as changes in their daily habits, including their habitual PA. Generally similar results were found in boys and girls - a decrease in PA over time, especially between 1992 and 2012, with a significant and negative trend for three of the four domains (household chores, games and walking).

In developing countries it is expected that children and adolescents be engaged in domestic tasks, spending a substantial part of their leisure time in these activities as part of their daily living [4]. For example, Filipino youth frequently reported that cooking and washing dishes and clothes were activities done after school/work [19], and in a study of Chinese youth, 11% of males and 20% of females reported several chores related to their housework as part of their daily PA [20]. The decrease observed in Mozambican youth’s household chores can be related to the economic and social transition that occurred during this 20-year period, which improved the living conditions of the population with increases in the percentage of houses with sanitation, tap water and electricity [21,22]. It is also associated with the availability/acquisition of new equipment and facilities to help their housework activities, which taken together in all likelihood contributed to the observed decline in opportunities to be physically active [23]. As such, children were less required to help in household chores, specifically in traditional activities listed in the questionnaire (cutting wood, pounding grain, agriculture, carrying water, cooking, cleaning, washing dishes, clothes washing). This trend was pointed out by Tudor-Locke *et al*. [19], that in developing countries, as modernization and technological advancement continue, primary sources of PA such as household chores, will decrease.

A significant negative trend was observed for the games domain in both boys and girls (1992 > 1999; 1992 > 2012). This result suggests that traditional games are being replaced by other behaviors during leisure time, such as TV viewing, computer or videogame use among Mozambican youth. Recent data pointed out that in developed countries a significant proportion of children’s leisure time is spent on “electronic entertainment” [24], with evidence that this behavior is also being observed among youth from developing nations [24,25,26]. Given that Mozambique has gone through relevant social and economic transitions, it is very well possible that these changes affected youth’s leisure time activities, allowing for less time spent in traditional games but increased time spent using electronic media [8]. 

There is no general consensus regarding recent secular trends in children’s and adolescents’ sports participation. For example, Westerstahl *et al*. [27] investigated Swedish youth and found an increase of their involvement in leisure-time sports activities over a 20-year period. Similarly, among Icelandic adolescents studied between 1992 and 2006, an increase in their active sports club participation was observed [28]. On the contrary, Mak and Day [29] reported a decrease in Hong Kong adolescents’ sports participation between 1995 and 2000. Additionally, Dollman, Norton and Norton [24] concluded that in many countries there has been a decline in youth involvement in organized sports. In Mozambican youth, results were similar in boys and girls, with statistically significant non-linear trends in both genders. We speculate that the observed reduction, in both genders, between 1999–2012 in the sport domain follows the tendency observed for household chores, and can be justified by the less time they spend in PA (structured or unstructured), and more time in sedentary leisure-time activities.

Walking has also shown a decline in the last 20 years among Mozambican youth. In the early 1990’s, it was usual that children and adolescents went to/from school by active means (*i.e.*, walking), as well as walking to perform other activities such as household chores. As such, walking was an important mode of “active” transportation for Mozambican youth [30]. Recent data showed that in the last decades a general decline in walking was observed among youth from different nationalities [31,32]. However, these studies focused mainly on the use of walking as transportation to/from school, not taking account its other use as a way of transport and as informal daily activity as it was done in the present study. A recent review with data from USA and Western European countries [33] confirmed a general decrease in walking (not only used as transportation to/from school) in different age groups and in both genders since 1970’s–1980’s. One explanation for this decline is related to the urbanization process with increases in automobile ownership (replacing active transport by motorized transport) [34]. Further, traffic danger [34], lack of available sidewalks [33], and street design [34] are also advanced as walking barriers. We favor that these factors, in association with other changes coming from the increased urbanization observed in Mozambique during the last 20 years (changes in household chores and household transportation options) are possible reasons for the observed decline in walking.

Since the AC is the unweighted sum of the four sub-domains, its decline is expected among Mozambican youth from 1992 to 2012. This result is in accordance with previous studies that reported PA declines in youth [6,24,34] as a consequence of their lifestyle change. For example, in Sub-Saharan African youth, a marked decline in PA was observed, which was associated with the urbanization process [6].

For most PA domains for boys (except for sports), as well as for games and walking domains for girls, no significant changes were observed between 1999 and 2012. We speculate that these results may be linked to the fact that in first decade after the War, the urbanization process had a greater impact on Mozambican lives, with fast changes in lifestyle such as the acquisition of new behavioral and nutritional habits. In the second decade (from 1999–2012) a stabilization of this process may have induced a reduction in these changes, with no more significant changes in youth’s PA habits.

This study provided relevant information about different PA domains in Mozambican youth. Yet, several limitations should be mentioned. First, the use of a questionnaire to estimate PA is prone to error, namely walking which can be underestimated when measured by a self-report instrument [19]. However, the use of a culturally validated questionnaire specifically for Mozambican children and adolescents, assessing different facets of their daily PA is a positive aspect. Second, dividing the sample into age groups could have provided more detailed information as it is known that children and adolescents have distinct PA levels and patterns. However, given the sample size, splitting it into different age groups would imply a reduction in statistical power. Third, no socio-economic or urbanization information was collected at the three time points. If we had such relevant information in all likelihood we would have a more thoroughly explanation about the impact of urbanization changes on youth’s PA. Fourth, information regarding sedentary behaviors was not collected, which would have provided an additional dimension to this study of changes in movement patterns among youth. Fifth, notwithstanding the quality of the Maputo sample, it is not representative of the entire country. Therefore the degree to which these results can be generalized to other regions is not known.

This study also has relevant strengths. First, it was conducted with a consistent and reliable data set from a developing country in Africa, covering an important transitional period. Second, the use of three time points allowed for a better understanding of trends in PA that occurred in Mozambique with the urbanization process after the end of the War. Third, the use of the same questionnaire to estimate PA that was applied on an individual-based interview by trained technicians at all time-points is an important strength. Fourth, the relatively large sample size covering an important lifetime period—childhood and adolescence, is another strength.

## 5. Conclusions

In summary, the present study showed a negative secular trend in Mozambican youth’s habitual PA, as well as a negative trend for most PA domains. These results suggest that observed changes in lifestyle and the environment in the last 20 years, as a consequence of socio-economic trends, may have had an important influence on children’s and adolescents’ PA. Future studies, investigating the casual links between socio-economical changes and PA in Mozambican youth, are needed to better clarify this issue. As the complex relationship among PA, overweight/obesity, physical fitness and health is unfolding, these results have important education and public health implications. Therefore, since more than 40% of the Mozambican population is under 15 years old [12,13], we suggest that different PA/physical education/sports participation programs should be implemented as a potential strategy to reduce the health risks associated with an inactive lifestyle in later life. Moreover, attention to the urbanization process must be done in order to reduce the impact of activity barriers in the growing cities.
